# Associations between physical and mental health and the utilization of ambulatory and emergency healthcare among asylum-seekers: results from a cross-sectional survey in Berlin, Germany

**DOI:** 10.1186/s12939-023-01914-6

**Published:** 2023-05-23

**Authors:** Nora Gottlieb, Martin Siegel

**Affiliations:** 1 Department of Population Medicine and Health Services Research, School of Public Health, Bielefeld University, PO Box 10 01 31, 33501 Bielefeld, Germany; 2 Department of Health Care Management, Technische Universität Berlin, Strasse des 17. Juni 135, 10623 Berlin, Germany; 3 Department of Empirical Health Economics, Technische Universität Berlin, Strasse des 17. Juni 135, 10623 Berlin, Germany

**Keywords:** Asylum-seekers, Chronic disease, Emergency care, Germany, Healthcare utilization, Mental health, Refugees, Structural equation model

## Abstract

**Background:**

Despite a high burden of chronic and mental illness, asylum-seekers show low utilization of ambulatory specialist healthcare. Forgoing timely healthcare when facing access barriers may direct them toward emergency care. This paper examines interrelations of physical and mental health and utilization of ambulatory and emergency care, and explicitly addresses associations between the different types of care.

**Methods:**

A structural equation model was applied to a sample of *n* = 136 asylum-seekers living in accommodation centers in Berlin, Germany. Utilization patterns of emergency care (outcome) and physical and mental ambulatory care (endogenous predictors) were estimated, while controlling for age, gender, chronic conditions, bodily pain, depression, anxiety, length of stay in Germany (exogenous predictors) and self-rated health (endogenous predictor).

**Results:**

Associations were observed between ambulatory care utilization and poor self-rated health (0.207, CI: 0.05; 0.364), chronic illness (0.096, CI: 0.017; 0.175) and bodily pain (0.019, CI: 0.002; 0.036); between mental healthcare utilization and anxiety (0.202, CI: 0.051; 0.352); and between emergency care utilization and poor self-rated health (0.621, CI: 0.059; 1.183), chronic illness (0.287, CI: 0.012; 0.563), mental healthcare utilization (0.842, CI: 0.148; 1.535) and anxiety (0.790, CI: 0.141; 1.438) (values in parentheses show estimated regression coefficients and 95% confidence intervals). We found no associations between the utilization of ambulatory and emergency care.

**Conclusions:**

Our study generates mixed results concerning associations between healthcare needs and ambulatory and emergency care utilization among asylum-seekers. We found no evidence that low utilization of ambulatory care contributes to emergency care utilization; neither did we find evidence that ambulatory treatment obviates the need to seek emergency care. Our results indicate that higher physical healthcare needs and anxiety are associated with more utilization of both ambulatory and emergency care; whereas healthcare needs related to depression tend to remain unmet. Both the undirected and under-utilization of health services may reflect navigation and accessibility issues. To facilitate more needs-based and effective healthcare utilization and thus contribute to health equity, support services such as interpretation and care navigation as well as outreach are warranted.

## Background

Despite substantial healthcare needs, asylum-seekers in Germany exhibit lower utilization of ambulatory specialist healthcare and ambulatory mental healthcare than statutorily insured persons [1–8]. At the same time, the incidence of emergency room visits and avoidable hospitalizations is comparatively high [1, 2, 4, 5, 7]. It has been suggested that these patterns are causally related, reflecting and reproducing inequities in healthcare and health: Formal and informal access barriers may make asylum-seekers forgo timely treatment in the ambulatory sector and instead use other, potentially inadequate and more costly emergency health services [4, 9–11]. Discrepancies between healthcare needs and the treatment provided, in turn, are liable to frustrate patients and care-givers [12, 13]. In the long run, inequitable treatment of physical and mental health conditions compromises the wellbeing, quality of life, and social integration of asylum-seekers and entails economic costs, for example through reduced productivity or resources spent on informal care [9, 14].

This paper investigates the associations between physical and mental healthcare needs and ambulatory and emergency healthcare utilization among asylum-seekers. It applies a Structural Equation Model (SEM) to data generated by a cross-sectional survey of asylum-seekers in Berlin. To our knowledge, this is the first paper that addresses the role of ambulatory physical and mental healthcare utilization for emergency care utilization by explicitly modeling potential interdependencies between the different types of care.

A note on terminology: we will use the term “forced migrants” in this paper to refer to various categories of displaced populations. With regard to the study population we will use the term “asylum-seekers”. However, our definition of the term is not in entire accordance with the respective administrative category, which exclusively denotes persons whose asylum claim is pending. For the purpose of this paper, we denote as asylum-seekers all persons whose social benefits are, de facto, regulated by the German Asylum Seeker Benefits Act, including persons who have submitted an asylum application, persons with renewable legal status such as a residence permit on humanitarian grounds, “failed” “non-deportable” asylum-seekers, and recognized refugees who, for any reason, have not yet access to full social entitlements [15].

### Healthcare needs of asylum-seekers

Forced migrants are at particular risk for physical and mental ill health because of structural factors before, during and after displacement [16–18]. Infectious diseases and, to a certain extent, mental trauma have been the primary foci of health system responses in transit and destination countries. Healthcare provision for displaced populations thus tends to overlook further healthcare needs such as services for chronic non-communicable diseases, sexual and reproductive health, or dental health [18, 19]. Especially chronic non-communicable diseases have been described as an emerging challenge in the context of recent forced migratory movements such as from Syria and Ukraine [20–22].

In the German context, comprehensive information on asylum-seekers’ healthcare needs is unavailable [23]. Empirical studies indicate that the prevalence of communicable diseases is comparable to the general German population [4, 24]. Regarding chronic non-communicable diseases, study results differ: Some authors report a relatively low prevalence of chronic illness among asylum-seekers, while stating that findings may be due to underreporting [6, 24]. Others show a similar or higher prevalence of chronic diseases as compared to the general population [5, 25]. Bauhoff and Göpffarth [4], for example, found prevalences of 20%, 48% and 62% respectively for nutritional anemia, diabetes and hypertension (as compared to 9%, 50% and 62% among the general population). A high prevalence of unspecific symptoms has been linked to psychological distress and somatization [4, 6, 24, 26]. This is consistent with reports of a high prevalence of mental illness among asylum-seekers in Germany: up to 77% for post-traumatic stress disorder [27] and 40–50% for depression and anxiety [4–6, 28] respectively. Studies consistently report overall low subjective health [5, 6].

### Accessibility of healthcare for asylum-seekers

In Germany, asylum-seekers have state-sponsored health coverage. Yet, like in many other host countries [19], restrictions apply to their scope of health entitlements (and to their access to healthcare, depending on their exact place of residence). During their first 18 months in the country, asylum-seekers’ health entitlements are limited to the treatment of acute, painful and life-threatening conditions, pregnancy and obstetric care, and vaccinations. Further medical services such as treatment of chronic diseases and mental health conditions can be covered based on an individual case review [15]. In theory, these regulations allow for a scope of health services that is near-equivalent to statutory health insurance. Yet, in practice, uncertainties over the correct interpretation of the law have led to inconsistencies in the authorization, provision and reimbursement of healthcare services for asylum-seekers, and eventually to a more restrictive implementation than justified from legal and medical perspectives [29]. For instance, in 2017, 49% of asylum-seekers’ applications for psychotherapy coverage were rejected after the individual case review, as compared to a rejection rate of 6% among statutorily insured persons [30]. Inconsistencies exist also in asylum-seekers’ access to health services, with different local authorities implementing different access models [2, 31]. In Berlin, which is the context of this study, asylum-seekers obtain an electronic health insurance card upon arrival, which, in theory, allows for healthcare access in a similar fashion as statutory health insurance [12].

In addition to formal barriers, asylum-seekers may experience various informal barriers. For instance, considerable communication barriers and sometimes negative attitudes from the part of administrative and medical staff have been reported [32–34]. They have been linked to insufficient coverage of medical interpretation services and general deficits in the accommodation of diversity in healthcare provision in Germany [35, 36]. In mental healthcare, specifically, a shortage of healthcare providers poses problems. Although specialized “Psychosocial Centers” (in German: Psychosoziale Zentren, PSZs) offer mental healthcare for asylum-seekers in addition to psychotherapists and psychiatrists, existing capacities cannot meet the demand [36]. In 2019, the PSZs reported waiting times of up to two years for psychotherapy and a 40% rejection rate due to a lack of capacities [30]. Stigma toward mental illness and healthcare creates additional hurdles [34, 37].

It has been hypothesized that the existing barriers to ambulatory specialist and mental healthcare incentivize asylum-seekers to seek emergency care and thus contribute to avoidable hospital visits. The goal of our study is to test this hypothesis by examining interrelations of physical and mental healthcare needs and the utilization of ambulatory and emergency care among asylum-seekers, while explicitly addressing associations between the different types of care.

## Methods

### Study design and sampling

This study used a cross-sectional survey on the health and healthcare utilization among asylum-seekers. The target population were residents of shared accommodation centers for asylum-seekers in Berlin, who were of legal age (18 years and above), and who were able to complete the questionnaire in one of the nine languages provided. Administering the survey in accommodation centers offers an opportunity to obtain a representative sample of the heterogeneous asylum-seeking population in Germany, because German law obliges asylum-seekers to reside in shared accommodation centers for the first 18 months of their stay or until obtaining permanent residency status. Given the scarcity of affordable housing, however, some asylum-seekers remain in shared accommodation centers beyond the designated period.

A clustered randomized sampling approach was applied to include a representative distribution of accommodation centers in the study: Using a complete list of Berlin’s accommodation centers, the facilities were divided into three categories, according to their capacity (small facilities with less than 250 persons, medium facilities with 250–500 persons, and large facilities with over 500 persons). The distribution of asylum-seekers across the different categories was calculated (19% in small centers, 59% in medium centers, 22% in large centers) and proportional numbers of accommodation centers were drawn from each category to achieve a similar distribution in the study sample.

Accommodation centers were contacted via email and telephone. If no contact could be established or participation in the survey was rejected, a new accommodation center from the same category was drawn. Within each participating accommodation center, the research team endeavored to approach and include the highest possible number of respondents.

### The questionnaire

The study used a shortened version of a questionnaire that had been developed for a different project (RESPOND, “Improving regional health system responses to the challenges of migration through tailored interventions for asylum-seekers and refugees”) [5]. Our version comprised 55 items including I) health status, II) healthcare utilization, and III) sociodemographic information.

I) Health status included self-assessed health status (measured on a Likert scale from 1 = very good to 5 = very poor), chronic illness (“Do you have any longstanding illnesses or health problems?”), screening items for depression (PHQ-2) and anxiety (GAD2), and a six-point scale for bodily pain.
II) Utilization of healthcare within the preceding 12 months (yes/no) was surveyed for general practitioners, specialist practitioners, psychotherapists and psychiatrists (in the following: mental healthcare providers), and emergency care. The number of emergency room visits in the last 12 months was assessed.
III) Sociodemographic information included gender (female/male/diverse), age, legal status (asylum application pending/asylum application concluded with refugee status/subsidiary protection/rejection and non-deportability/rejection), length of stay in Germany, and the highest level of formal education accomplished.

The questionnaire and all related information material were available in nine languages (Albanian, Arabic, English, Farsi, French, German, Russian, Serbian, and Turkish).

### Ethical approval

Ethical clearance was obtained from the Ethics Committee of the Charité University Clinic Berlin (EA4/111/18).

### Data collection

Data were collected between June 2018 and December 2019. The research team announced the study to staff and residents of the participating accommodation centers via posters and written information in different languages and, if possible, in person, for example during in-house plenary sessions. Depending on local conditions, the questionnaire was administered in two different ways: In some accommodation centers, the research team went from door to door and invited the residents to participate in the survey. In other centers, the team positioned itself in a public area of the accommodation center and invited passers-by to participate. In either case, symbolic giveaways (such as notice books, cosmetics, tea and biscuits) were offered irrespective of study participation. Study information was provided in writing, in the above said nine languages. Given the diverse composition of the research team, questions by potential participants could be answered in some, albeit not all languages (no professional translator was present). In addition, the team used audio recordings of the study information in five languages (Albanian, Arabic, Farsi, Kurdish, Russian) to help explain about the study. It tried to avoid any pressures to partake in the study, for example by emphasizing that participation would neither affect the asylum procedure nor involve any other personal benefits. The questionnaire was handed out on paper for independent completion, together with a stamped envelope. Filled-out forms were returned in three different ways: 90% were handed over in person, 5% were deposited in a closed box in the accommodation center, and the remaining 5% were sent by mail.

### Variables

The variables used for the analysis include binary variables for the presence of a chronic disease, symptoms of depression and symptoms of anxiety, utilization of ambulatory physical healthcare in the preceding 12 months (any ambulatory care physician excluding psychotherapists and psychiatrists), utilization of ambulatory mental healthcare (psychotherapists or psychiatrists) in the preceding 12 months, and less-than-good (i.e., very poor, poor or fair) self-assessed health. Bodily pain was included on a six-point scale. The number of emergency care visits in the preceding 12 months is the main outcome of this study.

### Statistical analysis

A Structural Equation Model (SEM) was employed to investigate the potential interrelations of physical and mental health, of ambulatory physical and mental healthcare utilization, and of emergency care utilization. The SEM allows for a simultaneous estimation of the associations among physical and mental healthcare needs and the utilization of different types of health services. This was considered necessary here because the different services are neither mutually exclusive nor independent from one another. A set of regressions with one-dimensional outcomes may therefore lead to biased results, when neglecting the expected correlations of ambulatory physical and mental healthcare utilization with the utilization of emergency care. The SEM further allows for modelling the associations between health and healthcare utilization as both direct and indirect effects. This was done here to examine how having accessed ambulatory physical and/or mental healthcare in the preceding 12 months influences emergency care utilization.

**Fig. 1:**
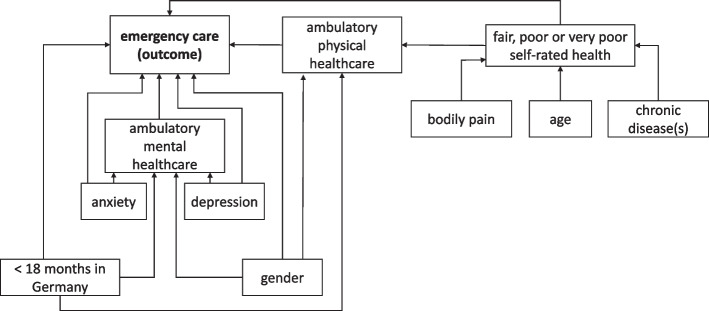
Full estimation model. The illustration shows our full estimation model, built of direct and indirect associations among physical and mental health, ambulatory physical and mental healthcare utilization, and emergency care utilization among asylum-seeking respondents

The estimation model shown in Fig. 1 is built as a set of direct and indirect associations between health and healthcare utilization. Poor self-assessed health is included as an endogenous predictor of ambulatory physical healthcare and emergency care utilization, with chronic illness, bodily pain and age serving as exogenous predictors of self-rated health. Symptoms of depression and anxiety are considered as exogenous predictors of ambulatory mental healthcare and emergency care utilization. Direct paths are included from ambulatory physical and mental healthcare utilization to emergency care utilization to assess whether ambulatory care utilization is associated with emergency care utilization. Indirect paths are modelled from poor self-rated health and from the presence of a chronic illness to ambulatory physical healthcare and emergency care utilization, as well as from the presence of depressive symptoms and the presence of symptoms of anxiety to emergency care utilization. Gender and less than 18 months of stay in Germany are included with direct effects on all types of healthcare utilization, to control for potential effects of the scope of health entitlements and familiarity with the German healthcare system on patterns of healthcare seeking. All estimations were performed in Stata 15.1 using the SEM command.

## Results

### Descriptive results

Twenty-two out of the 74 asylum-seeker accommodation centers in Berlin participated in this study. At the time, the total number of residents of asylum-seeker accommodation centers in Berlin was 6,399. Among them, an estimated 3,839 (60%) met the inclusion criteria for this study. Out of 811 persons who could be approached, 327 filled out and returned the questionnaire, which corresponds to a cooperation proportion of 39% and a response proportion of 8%. Upon data cleansing, a total *n* = 309 observations remained. Not all respondents completed all questionnaire items; our sample for a complete case analysis therefore comprises 136 observations.

Respondents were on average 34 years old; 37% described their gender as female and 63% as male. The average time since arrival in Germany was 39 months. 25% of the respondents arrived in Germany less than 18 month ago; their scope of healthcare entitlements therefore underlies restrictions. Almost half (46%) of the respondents described their physical health as less than good; 38% reported at least one chronic disease. Symptoms of at least one mental illness were reported by approx. 50% of the respondents. Thirty-nine percent of respondents reported depressive symptoms, 38% symptoms of anxiety. In total, 27% of the respondents reported symptoms of both anxiety and depression.

More than half of the estimation sample had had at least one ambulatory healthcare visit within the preceding 12 months. Only 21% of the respondents reported at least one mental healthcare visit in the previous year. One third of the respondents used emergency care at least once within the preceding 12 months; the average yearly number of emergency care visits among this group was 2.4. In the overall estimate sample, the average number of reported visits to an emergency room was 0.8. The descriptive results of the analysis are summarized in Table 1.

**Table 1:** Description of the estimation sample (*n* = 136)

		**Min**	**Max**
Mean age (in years)	33.9	18	68
Gender (female)	36.8%		
Fair, poor or very poor self-rated health	45.6%		
At least one chronic disease	37.5%		
Mean score of bodily pain (1 = low, 6 = high)	2.4	1	6
Symptoms of depression	39.0%		
Symptoms of anxiety	38.2%		
Mean length of stay in Germany (in months)	39.2	2	159
Share of respondents with stay < 18 months in Germany	25.0%		
Share of respondents who used ambulatory physical healthcare in the previous 12 months	55.9%		
Share of respondents who used mental healthcare (psychotherapy or psychiatry) in the previous 12 months	21.3%		
Average number of emergency care visits in the previous 12 months	0.8	0	10

### Estimation results

The likelihood-ratio-test indicates no significant difference between the observed and the estimated covariance structure of the data, suggesting that the estimated associations between health and healthcare utilization reflect the underlying interrelations. In addition, the Non-Normal (Tucker-Lewis) Fit Index is above 0.95 and the comparative fit index is above 0.9, both indicating a good model fit. The root mean squared error (RMSEA) of 0.034 is below the threshold of 0.05, which, too, suggests a good model fit. The model fit indexes are presented in Table 2.

**Table 2:** Goodness of fit indexes

Goodness of fit index	Value	Threshold
Likelihood-ratio-test (model vs. observed)	18.536 (*p* = 0.293)	*p* > 0.05
RMSEA	0.034	< 0.05
Comparative Fit Index	0.982	> 0.95
Tucker-Lewis (non-normal) Fit index	0.961	> 0.9

The estimates for the direct, indirect and total associations among the variables, together with 95% confidence intervals and associated *p*-values, are presented in Table 3. The structural equation for self-rated health suggests that bodily pain and the presence of a chronic disease are associated with poor self-assessed health. The estimated coefficients of chronic disease and pain are both positive, indicating that the risk to rate one’s health as fair, poor or very poor is higher if a respondent reports at least one chronic disease or bodily pain.

**Table 3:** Estimation results of the associations among health, ambulatory physical and mental healthcare utilization and emergency care utilization

	**Direct effect (coefficients)**			**Indirect effect (derived)**			**Total effects (derived)**		
	Coeff	95% CI	*p*-value	Effect	95% CI	*p*-value	Effect	95% CI	*p*-value
**Fair, poor or very poor self-rated health**									
Age	0.003	(-0.002; 0.009)	0.232				0.003	(-0.002; 0.009)	0.232
Chronic disease	0.463	(0.314; 0.612)	< 0.001				0.463	(0.314; 0.612)	< 0.001
Level of pain	0.091	(0.048; 0.134)	< 0.001				0.091	(0.048: 0.134)	< 0.001
Constant	-0.057	(-0.254; 0.140)	0.571						
**Ambulatory care utilization**									
Fair, poor or very poor self-rated health	0.207	(0.050; 0.364)	0.010				0.207	(0.050; 0.364)	0.010
Chronic disease				0.096	(0.017; 0.175)	0.018	0.096	(0.017; 0.175)	0.018
Level of pain				0.019	(0.002; 0.036)	0.029	0.019	(0.002; 0.036)	0.029
< 18 months in Germany	0.053	(-0.135; 0.241)	0.582				0.053	(-0.135; 0.241)	0.582
Gender	0.038	(-0.131; 0.208)	0.658				0.038	(-0.131; 0.208)	0.658
Age				0.001	(-0.001; 0.002)	0.278	0.001	(-0.001; 0.002)	0.278
Constant	0.437	(0.311; 0.563)							
**Mental healthcare utilization (psychiatry and/or psychotherapy)**									
Depressive symptoms	0.005	(-0.146; 0.156)	0.949				0.005	(-0.146; 0.156)	0.949
Symptoms of anxiety	0.202	(0.051; 0.352)	0.009				0.202	(0.051; 0.352)	0.009
< 18 months in Germany	0.138	(-0.015; 0.291)	0.078				0.138	(-0.015; 0.291)	0.078
Gender	0.066	(-0.071; 0.204)	0.345				0.066	(-0.071; 0.204)	0.345
Constant	0.075	(-0.031; 0.182)	0.167						
**Emergency care utilization**									
Ambulatory care utilization	0.388	(-0.171; 0.948)	0.174			0.041	0.388	(-0.171; 0.948)	0.174
Fair, poor or very poor self-rated health	0.541	(-0.028; 1.109)	0.062	0.080	(-0.051; 0.211)	0.229	0.621	(0.059; 1.183)	0.03
Chronic disease				0.287	(0.012; 0.563)	0.041	0.287	(0.012; 0.563)	0.041
Level of pain				0.056	(-0.001; 0.114)	0.055	0.056	(-0.001; 0.114)	0.055
Mental healthcare utilization	0.842	(0.148; 1.535)	0.017				0.842	(0.148; 1.535)	0.017
Depressive symptoms	-0.102	(-0.725; 0.520)	0.747	0.004	(-0.123; 0.131)	0.949	-0.098	(-0.733; 0.537)	0.762
Symptoms of anxiety	0.620	(-0.028; 1.267)	0.061	0.170	(-0.019; 0.359)	0.078	0.790	(0.141; 1.438)	0.017
< 18 months in Germany	0.021	(-0.582; 0.624)	0.945	0.136	(-0.053; 0.326)	0.159	0.157	(-0.463; 0.777)	0.619
Gender	-0.398	(-0.940; 0.145)	0.151	0.071	(-0.086; 0.227)	0.375	-0.327	(-0.889; 0.234)	0.254
Age				0.002	(-0.002; 0.006)	0.295	0.002	(-0.002; 0.006)	0.295
Constant	0.088	(-0.407; 0.582)	0.728						
**Error variances of endogenous variables**									
Fair, poor or very poor self-rated health	0.129	(0.102; 0.164)							
Ambulatory care utilization	0.231	(0.182; 0.293)							
Mental healthcare utilization	0.152	(0.120; 0.193)							
Emergency care utilization	2.304	(1.816; 2.922)							
Error covariance of ambulatory and mental healthcare utilization	0.059	(0.025; 0.092)							

Coefficients are direct effects; indirect effects and total effects are derived from estimated coefficients using the model structure. Effects are not necessarily causal

Only fair, poor or very poor self-rated health was directly and significantly associated with ambulatory physical healthcare utilization. Direct associations between less than 18 months in Germany and gender with ambulatory care utilization were small and close to zero when compared to the broad confidence intervals. Statistically significant indirect associations were found for chronic disease and bodily pain with ambulatory care utilization. The likelihood of ambulatory mental healthcare utilization is higher among respondents who screened positive for anxiety and for those who have been in Germany for less than 18 months, as compared to respondents who have stayed longer. (A sensitivity analysis that used legal status instead of length of stay yielded analogous estimates and a similar model fit.) The association of depressive symptoms with mental healthcare utilization is small and can be considered an imprecise zero with a *p*-value of 0.949.

Emergency care utilization yielded no clear associations with ambulatory physical healthcare utilization. In contrast, we found a positive association between emergency care utilization and mental healthcare utilization. Further variables that are likely to be associated with a higher frequency of emergency care utilization are chronic illness, bodily pain, and symptoms of anxiety. The presence of a chronic disease is indirectly associated with a higher frequency of emergency care utilizations, through its association with poor self-rated health. Likewise, bodily pain also has a significant indirect association with the utilization of emergency care services.

Having symptoms of anxiety shows a strong and significant positive association with the utilization of both ambulatory mental healthcare and emergency care. In contrast, symptoms of depression have no significant association with the utilization of neither ambulatory mental healthcare nor emergency care. Gender and length of stay exhibit no significant associations with emergency care utilization.

## Discussion

The aim of this study was to disentangle the interrelations between physical and mental healthcare needs, the utilization of ambulatory physical and mental healthcare services, and the utilization of emergency care by asylum-seekers. In light of formal and informal barriers to healthcare faced by this population, it has been suggested that foregone ambulatory healthcare visits may contribute to high emergency care utilization [4, 9, 11, 18]. Our study was the first to explicitly account for the non-exclusiveness and potential interdependence of the two types of care. The results indicate that ambulatory mental healthcare utilization, alongside poor self-rated health, chronic disease and anxiety, is significantly associated with an increased emergency care utilization. The utilization of ambulatory physical healthcare and the presence of depressive symptoms are not associated with emergency care utilization.

With regards to the question whether low utilization of ambulatory healthcare drives emergency care use among asylum-seekers, our study thus yields mixed results, which may reflect differences in healthcare seeking for different health problems. Overall, we found no significant negative associations between the utilization of any (physical or mental) ambulatory healthcare and emergency care utilization, as we would expect either a) if foregoing necessary visits to an ambulatory healthcare provider would eventually result in (avoidable) emergency room visits; or b) if health problems were resolved in ambulatory care, thus obviating the need for a visit to the emergency room.

As regards mental health, our results confirm discrepancies between asylum-seekers’ high burden of illness and their relatively low utilization of ambulatory mental health services, which have been reported in the international literature [38–40]. Our study indicates that asylum-seekers with depressive symptoms may be at particular risk of remaining underserved: The finding that depressive symptoms are not associated with the utilization of any type of healthcare points toward unmet treatment needs for this condition. Anxiety, on the contrary, is associated with an increased likelihood of using both ambulatory mental healthcare and emergency care. The positive association between the utilization of both ambulatory mental healthcare and emergency care may indicate that asylum-seeking patients tend to go back and forth between the two types of service, with neither one meeting their need. Our study thus supports previous claims concerning mismatches between asylum-seekers’ mental healthcare needs and the treatment provided for them [8, 28], with potential negative impacts on patients and healthcare systems.

As regards physical health, we found higher utilization of both ambulatory and emergency healthcare among asylum-seekers with higher physical healthcare needs. This could indicate a desirable outcome from the perspective of vertical health equity. However, it has been pointed out that high utilization of emergency care, in combination with frequent emergency room visits for non-severe conditions, may rather reflect accessibility issues or failures of ambulatory treatment to meet patients’ needs [4, 18]. It has been argued, for example, that excessive painkiller prescriptions for asylum-seeking patients signal a tendency to relief symptoms instead of endeavoring to cure the problem [3, 41]. Our findings lend only limited support for these claims (as we would then expect to find a negative association between ambulatory and emergency care utilization in case of inaccessibility, or a positive association in case of a mismatch of healthcare needs and provision). Another explanation may be that difficulties in navigating a bureaucratic, complex and fragmented healthcare system, in combination with a lack of understandable health information [6, 32], amke asylum-seeking patients rather “randomly” seek either ambulatory healthcare, emergency care, or both types of care to resolve physical ailments. Overall, our results underline that, to develop a nuanced understanding of health needs and healthcare-seeking among asylum-seeking populations, future research needs to include asylum-seekers’ perspectives [18, 19].

Similar to previous studies [42], we found that respondents who had arrived in Germany within 18 months before the survey were more likely to report utilization of mental healthcare. Newly arrived asylum-seekers not only face eligibility restrictions, but they are arguably less familiar with the German healthcare system. On the other hand, the coverage of medical interpretation services during the first 18 months of stay in Germany facilitates access to mental healthcare. Yet, this entitlement expires after 18 months [6, 15]. Another explanation for our finding is that the centralized accommodation of asylum-seekers during the first 18 months – despite its manifold disadvantages – may enable the provision of social support, including encouragement to seek mental healthcare and help with care navigation. Recent research on asylum-seekers’ perspectives on mental healthcare provision in Germany suggests that support services via official (e.g., social workers) and unofficial channels (e.g., volunteers and peers) play a key role in facilitating access to mental healthcare [34]. Asylum-seekers who move out of accommodation centers may be at risk of dropping out of these support networks. This indicates the need for institutionalized and expanded support services, which can facilitate needs-based healthcare seeking and continuity of care in a comprehensive and sustainable fashion [43]. The recent displacement of large numbers of persons from Ukraine and their decentralized settlement across the member states of the European Union renders this need all the more urgent. Ultimately, such change has the potential to make the healthcare system more accessible and responsive for diverse population groups, including asylum-seekers, and thus contribute to better, more equitable health system outcomes for all [44, 45].

This study has three major limitations. First, the sample size is comparatively small. This limits the generalizability of the results and leads to low statistical power, which, in turn, may result in insignificant results even if a systematic association exists. However, the sample size is sufficiently large for the employed SEM; and we consider the final estimation sample of 136 complete cases to be a good success in data collection, given the challenges in conducting empirical research with asylum-seeking populations [46]. We acknowledge that the small sample size may lead to the acceptance of mis-specified models, but the high Tucker-Lewis fit index (particularly recommended for small sample sizes) in combination with the likelihood-ratio-test suggests that the model is likely to describe the utilization patterns in the data. Second, the questionnaire asked for utilization of ambulatory healthcare, ambulatory mental healthcare, and emergency care over a period of 12 months, which may involve recall bias. However, we expect the potential range of errors to be rather small and, in addition, erroneously high and low responses to balance out, resulting in an overall low potential error. Third, the study was restricted to the German city-state of Berlin. Availability of interpretation services and healthcare providers with compatible language skills as well as healthcare provision within the first 18 months of stay vary considerably across Germany, thus limiting the generalizability of our findings. However, given the similarity of research findings from different contexts, the results of this study may provide some general insights into asylum-seekers’ healthcare needs and healthcare seeking.

## Conclusions

We found no clear evidence that low ambulatory healthcare utilization drives high emergency care utilization among asylum-seekers. However, our study indicates that some asylum-seeking patients go back and forth between ambulatory and emergency care, or seek either type of service “at random”. Asylum-seekers with depressive symptoms are at particular risk of remaining underserved. Both the undirected healthcare-seeking and the under-utilization of healthcare may reflect care navigation and accessibility issues. Our study thus lends support to previous claims concerning the German healthcare system’s deficits in terms of accessibility and responsiveness of service provision for asylum-seeking populations. To develop a nuanced understanding of the needs and healthcare seeking of asylum-seekers with chronic and mental illness, better data and further research, especially studies that include asylum-seekers’ perspectives, are required. Improving accessibility and responsiveness of healthcare services, including the expansion of institutionalized support, outreach, and coverage of interpretation services beyond asylum-seekers’ first 18 months in the country, may be key to improving health system outcomes, including more equitable and efficient healthcare provision for a diverse society.

## Data Availability

The instruments and datasets used and/or analyzed during the current study are available from the corresponding author and/or the RESPOND research team upon reasonable request.
